# Delivery of the Radionuclide ^131^I Using Cationic Fusogenic Liposomes as Nanocarriers

**DOI:** 10.3390/ijms22010457

**Published:** 2021-01-05

**Authors:** Rejhana Kolašinac, Dirk Bier, Laura Schmitt, Andriy Yabluchanskiy, Bernd Neumaier, Rudolf Merkel, Agnes Csiszár

**Affiliations:** 1 Institute of Biological Information Processing: Mechanobiology (IBI-2) Forschungszentrum Jülich GmbH, 52428 Jülich, Germany; rejhanakolasinacfzj@gmail.com (R.K.); l.schmitt@fz-juelich.de (L.S.); r.merkel@fz-juelich.de (R.M.); 2 Institute of Neurosciences and Medicine: Nuclear Chemistry (INM-5) Forschungszentrum Jülich GmbH, 52428 Jülich, Germany; d.bier@fz-juelich.de (D.B.); b.neumaier@fz-juelich.de (B.N.); 3 Center for Geroscience and Healthy Brain Aging, Department of Biochemistry and Molecular Biology, University of Oklahoma Health Sciences Center, Oklahoma City, OK 73104, USA; Andriy-Yabluchanskiy@ouhsc.edu

**Keywords:** cationic liposomes, fusogenic liposomes, radioisotope delivery, ^131^I, cancer

## Abstract

Liposomes are highly biocompatible and versatile drug carriers with an increasing number of applications in the field of nuclear medicine and diagnostics. So far, only negatively charged liposomes with intercalated radiometals, e.g., ^64^Cu, ^99m^Tc, have been reported. However, the process of cellular uptake of liposomes by endocytosis is rather slow. Cellular uptake can be accelerated by recently developed cationic liposomes, which exhibit extraordinarily high membrane fusion ability. The aim of the present study was the development of the formulation and the characterization of such cationic fusogenic liposomes with intercalated radioactive [^131^I]I^−^ for potential use in therapeutic applications. The epithelial human breast cancer cell line MDA-MB-231 was used as a model for invasive cancer cells and cellular uptake of [^131^I]I^−^ was monitored in vitro. Delivery efficiencies of cationic and neutral liposomes were compared with uptake of free iodide. The best cargo delivery efficiency (~10%) was achieved using cationic fusogenic liposomes due to their special delivery pathway of membrane fusion. Additionally, human blood cells were also incubated with cationic control liposomes and free [^131^I]I^−^. In these cases, iodide delivery efficiencies remained below 3%.

## 1. Introduction

Liposomes are widely used in pharmacology as carrier particles [1,2,3,4,5,6]. They are highly biocompatible, biodegradable, and non-immunogenic lipid-based vehicles suitable for the solubilization of hydrophobic and hydrophilic substances. Such drug carriers improve therapeutic efficacy by minimizing the rapid degradation of the cargo and increasing its absorption [7,8]. They are well established in cancer chemotherapy [9,10] and gene therapy [2]. In addition, since the late 1990s they have found broad application in nuclear medicine and diagnostics. In particular, image-guided drug delivery exploits the benefits of liposomal carriers. Due to the negative charge of natural phospholipids, liposomes intended for theranostic purposes usually intercalate radiometals, such as ^64^Cu, ^99m^Tc, ^188^Re, or ^89^Zr [11,12]. Loading efficiency was increased by click labeling or surface chelation [13]. Labeling of the liposomes with radionuclides containing pharmacologically active compounds enabled the in vivo tracking of the transport, accumulation, and clearance of the liposomes from the organism. For non-invasive imaging, positron emission tomography (PET), single-photon emission computed tomography (SPECT), and magnetic resonance imaging (MRI) were applied [11,12].

Radiolabeling of liposomes with neutral or negative surface charge has been extensively investigated over the past few decades. In contrast, studies using cationic liposomal carriers in conjunction with radionuclides are so far scarce. Such liposomes are popular in therapeutic gene delivery where the cargo DNA or mRNA forms stable complexes with the carrier particles due to attractive electrostatic interactions. Cationic liposomes are usually composed of the classical lipid mixture of the neutral lipid 1,2-dioleoyl-sn-glycero-3-phosphoethanolamine (DOPE) and the cationic lipid 1,2-dioleoyl-3-trimethylammonium-propane (DOTAP). As our previous research showed, the addition of aromatic molecules to the classical mixture at a concentration of 5 mol% or above renders cationic liposomes with extraordinary high fusion potential inducing their direct fusion with the cellular plasma membrane [14,15]. As a consequence, their cargo is delivered into the cytoplasm of the mammalian cells with high efficiency bypassing cargo degradation in the endosomes [16,17], which is considered to be the main liposomal uptake route.

Using such liposomes as carrier particles, the best cargo delivery was achieved when cargo internalization was based on the attractive interaction between the positively charged liposomal surface and the negatively charged cargo, e.g., proteins, peptides [17] or RNA molecules [18]. Therefore, we hypothesized that cationic liposomes effectively bind anionic radionuclides as well and the radionuclides are delivered to mammalian tissues and cells of interest. For example, free anionic iodide predominantly accumulates in the thyroid [19,20,21], whereas liposomes are mainly accumulated in tumor tissues due to their high metabolic activity [22]. If liposomes can effectively bind radioiodine, its targeting to non-thyroidal tissues becomes possible.

Accordingly, the present study aimed at the preparation and characterization of cationic fusogenic liposomes radiolabeled with [^131^I]I^−^. The epithelial human breast cancer cell line MDA-MB-231 was used as a model for invasive cancer cells. The cellular uptake of liposomal [^131^I]I^−^ was determined in vitro, and delivery efficiency was examined. Moreover, fusion with human blood cells was additionally analyzed since in a potential application liposomes will be injected into the blood stream.

## 2. Results

### 2.1. Characterization of Liposomes Containing Iodide as Cargo

**Figure 1:**
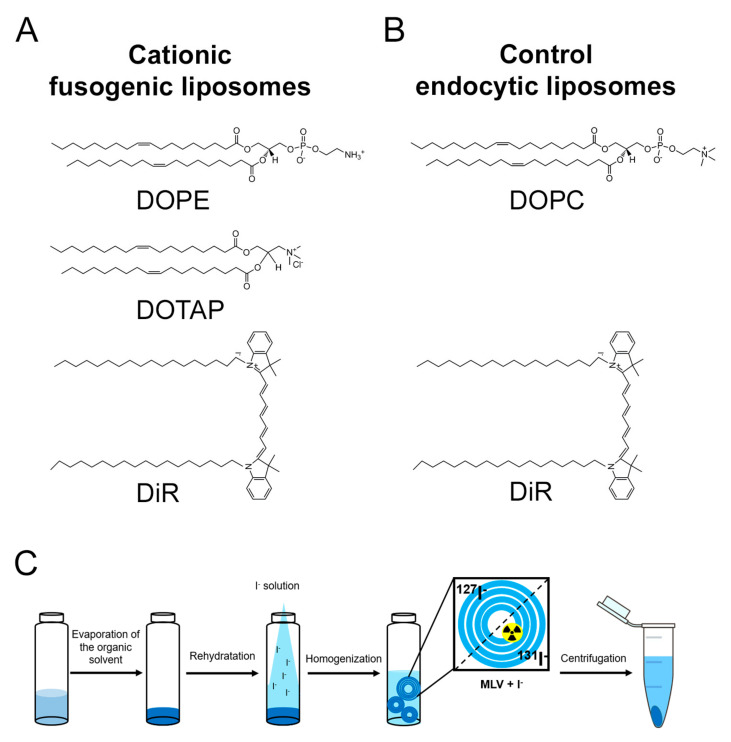
Chemical structures of the lipid molecules forming cationic fusogenic (**A**) and control endocytic liposomes (**B**). Preparation of iodide-loaded liposomes (**C**). After evaporation of the organic solvent, the dry lipid mixture was rehydrated in an iodide-containing buffer whereby liposomes containing iodide isotopes formed spontaneously between the lamellas. Iodide loading efficiency was determined on liposomes separated from the free solution by centrifugation.

Cationic fusogenic liposomes (FL) were prepared from the classical lipid mixture of the neutral lipid DOPE and the cationic lipid DOTAP (for IUPAC names see Materials and Methods Section). To visualize these liposomes and to render them fusogenic, the fluorescent lipid tracer DiR was added (1/1.2/0.3 mol/mol) (Figure 1A). The cationic lipid mixture was rehydrated in iodide solution at high lipid concentration without any additional homogenization steps apart from vigorous mixing (see Materials and Methods Section and Figure 1C).

**Figure 2:**
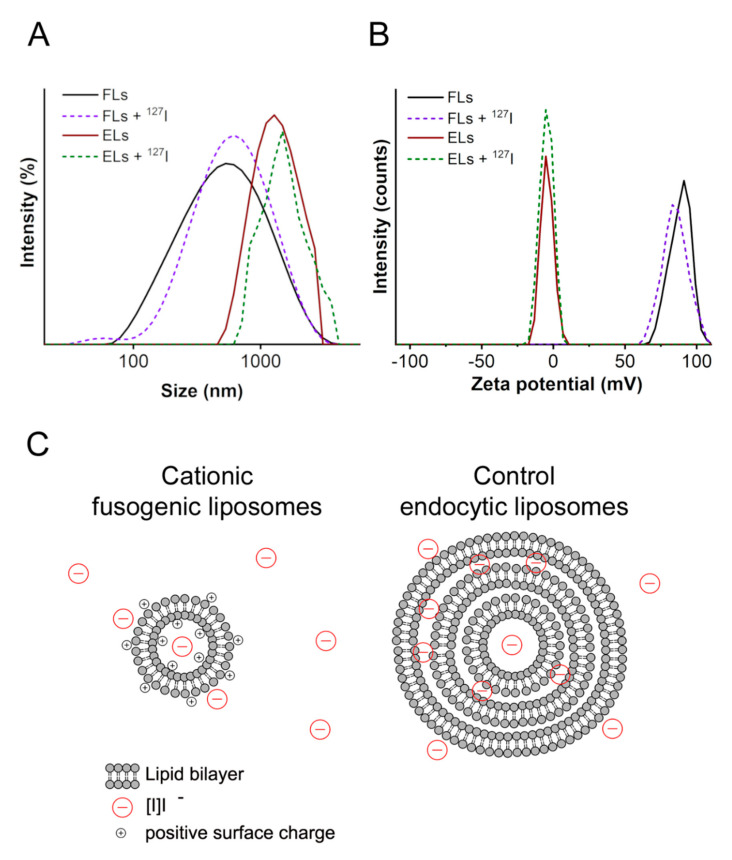
Representative curves of the hydrodynamic diameter (**A**) and zeta potential distributions (**B**) of cationic fusogenic (FL) and control endocytic liposomes (EL) with and without [^127^I]I^−^ incubation. Hypothetical structures of the same liposomes based on the physicochemical characterization (**C**). Note that the same liposomal structures are hypothesized in the presence of both [^131^I]I^−^ as well as [^127^I]I^−^.

For characterization purposes, the radionuclide [^131^I]I^−^ was replaced by the non-radioactive isotope [^127^I]I^−^ assuming that both isotopes interact with liposomal membranes in the same manner. The multilamellar liposomes formed had an average diameter of 456 (51) nm, which is slightly larger than liposomes without cargo (376 (5) nm). The polydispersity indexes (PDI) in both cases indicated similar size distributions. A representative distribution curve is shown in Figure 2A, while the statistical analysis of all executed measurements is summarized in Table 1. The small size of spontaneously formed liposomes was presumably stabilized by the strong electrostatic repulsion between the charged liposomes. Zeta potentials, characteristic of the electrostatic properties of the membrane surface of both [^127^I]I^−^ loaded and empty, liposomes were +89 (3) mV and +88 (5) mV, respectively, without any significant difference (Figure 2B and Table 1).

As a control sample, liposomes made of the natural phospholipid DOPC (for IUPAC name see Material and Methods) and the same fluorescent lipid tracer DiR (1/0.0025 mol/mol) were used (Figure 1B). Due to the neutral zeta potential of the control liposomes (−2.5 mV), they formed large liposomes and liposomal aggregates with an average size of approximately 2 µm and very high polydispersity (Figure 2A and Table 1). While iodide internalization decreased, the sample polydispersity, the zeta potential of the complexes remained unchanged (Figure 2B and Table 1).

Stability analysis of the complexes was carried out by monitoring size, PDI, and zeta potential changes over time. Here, a short time period of 10 days was chosen as investigation time based on the half-life of [^131^I]I^−^ of 8 days. As shown in Table 2, cationic liposomes, with or without [^127^I]I^−^ cargo underwent no significant changes over 10 days while both types of control liposomes remained highly heterogenous in size (see also PDI) with strongly altering zeta potentials.

**Figure 3:**
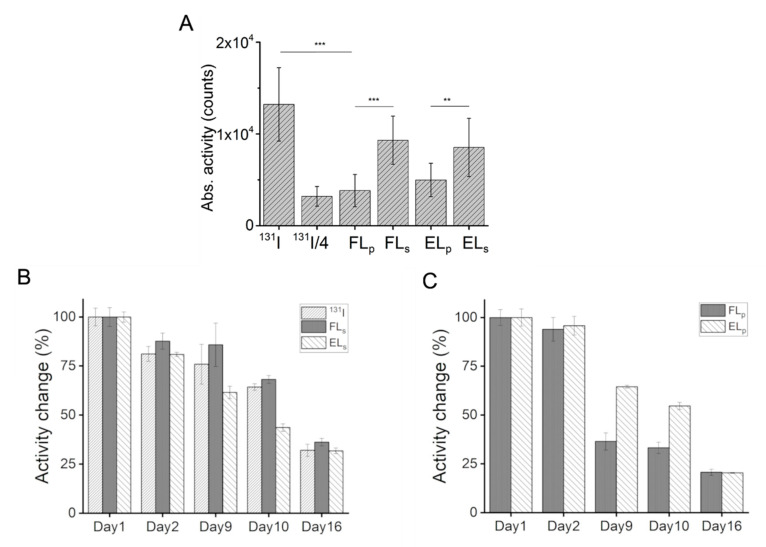
Absolute activity of free [^131^I]I^−^ radionuclide solution, as well as the activity of [^131^I]I^−^ intercalated into cationic fusogenic (FL) and control endocytic liposomal (EL) pellet (index p) and the activity of free [^131^I]I^−^ in the supernatant (**A**). The uptake efficiency of free [^131^I]I^−^ was determined using two different iodide concentrations, the initial one (^131^I^−^), and one quarter of this concentration (^131^I^−^/4), which was approximately identical to the amount of intercalated iodide in FLs. Activity changes of [^131^I]I^−^ in phosphate buffered saline (PBS) and liposomal supernatants (**B**) as well as in liposomal pellets (**C**) were monitored over 16 days. Statistical significances were considered as follows: *p* < 0.001 (***), *p* < 0.01 (**).

To determine iodide incorporation efficiency, liposomes intercalating the radionuclide [^131^I]I^−^ were prepared and the cargo-loaded particles were separated from the buffer by centrifugation. ^131^I-activities were determined in the pellet and the supernatant. Intercalation efficiency was calculated as the ratio of activity of pellet to total activity. As shown in Figure 3A, cationic liposomes were able to incorporate 29 (13)% and control liposomes 38 (14)% of the applied [^131^I]I^−^. Therefore, an additional control sample at 25% of the original iodide concentration was also applied in further in vitro experiments.

Because chemical interaction can influence [^131^I]I^−^ half-life (t_1/2_), the activity changes of [^131^I]I^−^ were monitored in all samples. As shown in Figure 3B, the measured activities in the PBS buffer and in both supernatant samples decreased linearly over time. The calculated half-lives of [^131^I]I^−^ did not differ from each other statistically (Table 3). In both liposomal samples, reduced half-lives were measured (Figure 3C and Table 3) compared to the supernatant samples. The values determined in liposomal pellets were statistically identical.

### 2.2. Incubation of Different Cell Types In Vitro Using Iodide-Loaded Cationic Liposomes

**Figure 4:**
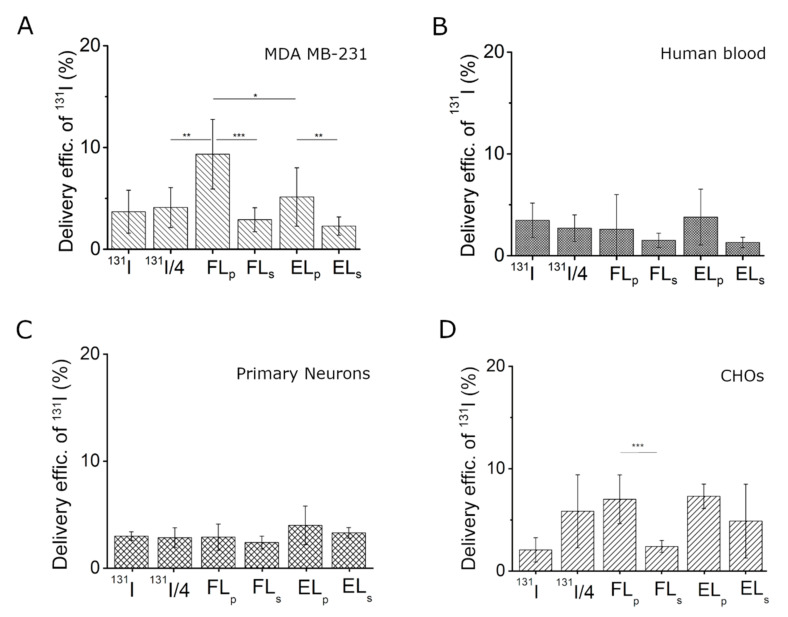
Delivery efficiencies of [^131^I]I^−^ using liposomes as carrier particles and free [^131^I]I^-^ from supernatants and neat solutions as controls to MDA MB-231 cancer cell line (**A**), human blood cells (**B**), primary neuronal cells (**C**), and Chinese hamster ovary (CHO) cells (**D**). Data are presented as mean (SD). N = 3. Statistical significances were considered as follows: *p* < 0.001 (***), *p* < 0.01 (**), *p* < 0.05 (*).

Iodide transfer efficiency was evaluated using the human epithelial breast cancer cell line MDA-MB-231 and cationic fusogenic liposomes or neutral control liposomes as carrier particles. The cargo-loaded particles were separated from free iodide by centrifugation. Although control liposomes were able to incorporate more [^131^I]I^−^ than cationic fusogenic liposomes, their delivery efficiency to the cancer cells remained only approximately one half of that achieved by cationic liposomes (5.1 (2.9)% vs. 9.3 (3.4)%, respectively) (Figure 4A). The uptake efficiency of free [^131^I]I^−^ was determined using two different iodide concentrations, the initial one, and one quarter of this concentration ([^131^I]I^−^/4), which was approximately identical to the amount of intercalated iodide in FL. Free iodide from liposomal supernatants was also analyzed. In all cases, iodide uptake was lower compared to liposomal delivery, and remained below 4% (Figure 4A).

**Figure 5:**
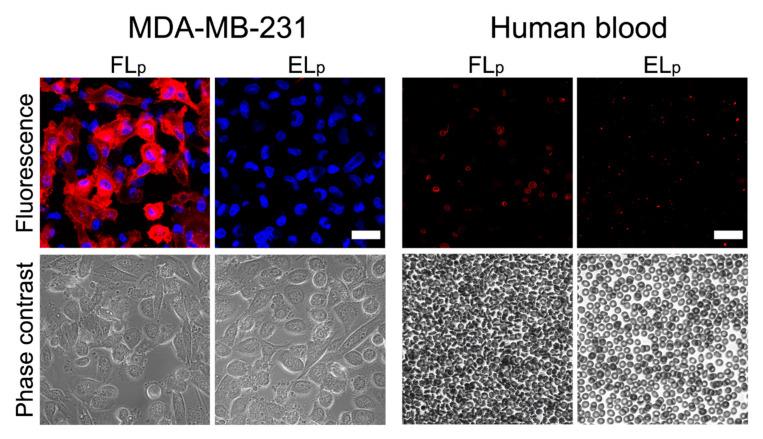
Monitoring the incorporation of cationic fusogenic (FL) and control endocytic liposomal (EL) pellet (index p) and supernatant (index s) containing [^127^I]I^−^ into MDA MB-231 cancer cells and human blood cells by microscopy. The fluorescence signal of the lipid tracer DiR (red) and the cell nuclei staining of DAPI (blue) are shown in overlays. Phase contrast images reveal healthy cell morphologies. Scale bars, 20 µm.

The best cargo delivery efficiency was obtained for cationic FL as nanocarriers. We assumed that this high value was achieved due to the different uptake mechanisms of these liposomes compared to the control. To test our hypothesis, the cellular uptake of cationic fusogenic and control liposomes was visualized using confocal microscopy by observing the fluorescence signal of the liposomal membrane tracer DiR. Cells were identified upon DAPI staining of their nuclei. As shown in Figure 5, DiR fluorescence was located mainly in the plasma membrane of MDA-MB-231 cells upon treatment with FL, and staining efficiency reached 90%. Compared to this, only a few dot-like signals of the lipid tracer DiR were detected on the cell surfaces treated with control liposomes. The liposomes were internalized first after several hours and became visible in the nuclei focal plane.

In the case of in vivo application of liposomes loaded with radioactive tracers, the complexes are directly injected into the bloodstream. Therefore we also analyzed [^131^I]I^−^ delivery to human blood cells in vitro. To this end, human blood samples were incubated with free and liposomal ^131^I at the same concentration as used on cancer cells and the cellular iodide activities were determined. As shown in Figure 4B and Figure 5, iodide delivery efficiencies remained comparatively low (approximately 3 (2)%) in all cases irrespective of treatment strategy.

We tested additional cell types for delivery efficiency of [^127^I]I^−^. Here, an epithelial cell line derived from the ovary of the Chinese hamster (CHO), as well as primary rat embryonic neuronal cells were chosen based on their elevated negative surface charges. Cells were treated with the same liposomes and the [^127^I]I^−^ activity was monitored. None of the treatments showed significantly increased [^127^I]I^−^ activity in those cell types as presented in Figure 4C,D.

## 3. Discussion

Cationic liposomes are mainly used for the transport of nucleic acids into living cells [23,24]. Here, a special type of such carriers, known as fusogenic liposomes, was used for the incorporation of ^131^I into breast cancer cells in vitro to evaluate the potential for application not only in gene therapy but also in radiotherapy.

The preparation method of the liposomes, the rehydration of the dry lipid film in a buffer solution containing iodide, as well as the separation of the cargo-loaded liposomes from the free solution by centrifugation are well established, and have been described elsewhere [25]. Surprisingly, cationic FL cannot be separated from the supernatant by centrifugation at room temperature. A decrease of temperature from 20 °C to 4 °C resulted in an efficient separation of the liposomes from the free buffer, presumably due to the formation of the lamellar lipid phase at low temperatures. Due to the high positive charge of the lipids, a stable liposomal suspension with an average particle size of 400 nm was formed [26] with a moderate iodide encapsulation efficiency of 29%. The main benefit of such liposomes is their long-term stability (Figure 2C). As a control, liposomes with neutral charge were used. Such liposomes are stable only for 1–2 days and lose the main part of their cargo afterwards. Moreover, additional preparation technique is needed for decreasing the liposomal size from µm- to nm-range. This high incorporation efficiency of control liposomes (38%) can presumably be attributed to the high amount of iodide-containing buffer intercalated between the lamellas of the µm-sized liposomes. Compared to this, cationic FL were found to be 4−5 times smaller (see Table 1) and probably formed from much fewer alternating lipid/water layers. As shown elsewhere, such liposomes have only partially lamellar structures [27] and contain less water. Therefore, the amount of cargo dissolved in buffer is reduced. A schematically representation of both liposomal structures is shown in Figure 2C. 

Based on our analysis, we state here, that the different liposomal structures of the investigated liposomes prolonged the ^131^I half-life in the same manner (t_1/2_ 10–11 days) compared to free ^131^I (t_1/2_ 8 days) [28]. It is known that the half-life of radioactive decay can be altered by changing the state of the electrons surrounding the nucleus. Since the chemical bonding between atoms involves the deformation of atomic electron wave functions, the radioactive half-life of an atom can depend on the atomic bonds [29]. Our results clearly indicate the presence of a chemical bond of ^131^I to both lipid membranes.

The cationic FL provided the highest ^131^I delivery efficiencies into triple-resistant breast cancer cells (MDA MB-231). In all other cases, iodide uptake remained lower (see Figure 4B–D). We assume that the high ^131^I concentration caused high cell toxicity as well, although cell viability has not been proven in this study. The result is in agreement with other reports about the very low accumulation of ^131^I in non-thyroidal tissues [30]. The high delivery efficiency by using FL was probably achieved due to the different uptake mechanism of these liposomes compared to the control. As previously shown, cationic FL containing sufficient amounts of aromatic molecules are able to fuse with the plasma membrane of mammalian cells, whereby they deliver their cargo with high efficiency directly into the cell interior [17]. Here, the best loading efficiencies were achieved using negatively charged cargos due to the attractive electrostatic forces between cargo and carrier particles. Negatively charged proteins, e.g., eGFP or phycoerythrin [17], as well as nucleic acids (e.g., mRNA, siRNA) [18], have been successfully tested.

As our results showed, the anionic [^131^I]I^−^ can also be efficiently delivered by cationic FL. We assume that, similar to proteins and RNA mentioned above, the negatively charged iodide ions are also bound to the cationic liposomal surface (Figure 2B) and are directly transported to the breast cancer cells via membrane fusion thus achieving high delivery efficiencies. However, cancer cells in general, have an overall negative surface charge and can be targeted by cationic nanoparticles very efficiently based on the attractive electrostatic forces between particle and cell membrane surfaces [31,32], we focus our statement exclusively on the breast cancer cell line used in this study.

In the case of therapeutic applications, liposomes are usually directly injected into the bloodstream and are transported to different organs and tissues [33]. Their direct absorption by blood cells is not desirable with the exception of some special blood treatments [34]. Therefore we analyzed [^131^I]I^−^ delivery to human blood cells as well. As shown in Figure 3C and Figure 4, iodide delivery efficiencies remained comparatively low (approximately 3 (2)%) in all cases irrespective of treatment strategy. These results are especially surprising in the case of FLs because they interact directly upon contact with almost all kinds of mammalian cells, including purified red blood cells [35], and fuse with their plasma membranes. We hypothesize that serum proteins might be able to block the fusion process between liposomes and blood cell [36] complexes for intravenous gene delivery (Figure 5). The role of the protein corona forming on nanoparticle surfaces has been previously described [37,38]; however, the formation of such complexes containing cationic FL and serum proteins needs further investigation. In the case of protein/lipid complex formation, FL also remain adherent to the plasma membrane, similar to EL, and are intercalated by endocytosis instead of membrane fusion. As already shown, this uptake route is less beneficial for molecular delivery applications [18]. As our previous results showed, the complex formation of cationic FL with serum proteins after a retro-orbitally injection into mice is a temporary process [39]. Here, the liposomes were able to be released from the complex after accumulation in the endothelium of blood vessels. Therefore, we hypothesize that liposomal transport into different tissues become possible after disassembly of the protein corona on the liposomal surface.

## 4. Materials and Methods

### 4.1. Chemicals

Fusogenic liposomes were prepared using the cationic lipid 1,2-dioleoyl-3-trimethylammonium-propane (chloride salt) (DOTAP) and the neutral lipid 1,2-dioleoyl-sn-glycero-3-phosphoethanolamine (DOPE) while control liposomes were prepared by using only the neutral lipid 1,2-dioleoyl-sn-glycero-3-phosphocholine (DOPC). All above-mentioned lipids were purchased from Avanti Polar Lipids, Inc. (Alabaster, AL, USA) and used without further purification. As a fluorescent tracer, the lipid analogue 1,1′-dioctadecyl-3,3,3′,3′-tetramethylindotricarbocyanine iodide (DiIC_18_(7) also known as DiR (Thermo Fisher Scientific Inc., Eugene, OR, USA) was incorporated into both types of liposomes. Non-radioactive [^127^I]I^−^ (ACS reagent, ≥99.5% from Sigma Aldrich, Taufkirchen, Germany) as well as radioactive [^131^I]I (iodide radionuclide 185 MBq (5 mCi), in 0.1 M NaOH (pH 12–14)) from PerkinElmer, Hamburg, Germany)) were used as sodium salt. The nuclei-staining blue fluorescent dye 4′,6-diamidino-2-phenylindole (DAPI) was purchased from Thermo Fischer Sci. (Eugene, OR, USA).

### 4.2. Liposomal Preparation and Characterization

#### 4.2.1. Preparation of Liposomes Containing Iodide

Fusogenic liposomes were prepared by mixing the neutral lipid DOPE and the cationic lipid DOTAP with the fluorescent DiR in chloroform (EMSURE grade, VWR, Darmstadt, Germany) in a ratio of 1/1.2/0.3 mol/mol. For control liposomes, the neutral lipid DOPC and the fluorescent tracer DiR were mixed in a molar ratio of 1/0.0025 mol/mol. Chloroform was evaporated under vacuum for 30 min. Subsequently, the dry lipid films were hydrated using either 20 µL of sucrose solution with [^131^I]I^−^ (3.7 kBq/µL, 160 mOsm/kg) or 20 µL of sucrose solution with non-radioactive [^127^I]I^−^. The iodide concentrations were set to 0.9 pg/µL in both solutions. Samples were vortexed for 20 min until the lipid film was completely hydrated. The lipid concentration in both cases was set to 4 mg/mL. Subsequently, liposomes were diluted with 250 µL of sucrose solution (160 mOsm/kg) and stirred for an additional 10 min before being divided into two parts and 1 mL of cold phosphate buffered saline (PBS) (Thermo Fisher Scientific Inc., Eugene, OR, USA) added to each part. The solutions were centrifuged at 4 °C for 30 min at 25,000× g (centrifuge 5417R from Eppendorf, Wesseling-Berzdorf, Germany). After centrifugation, the supernatant was separated from the pellet (approximately 90 µL pellet).

For the preparation of control samples, the same amount of [^131^I]I^−^-solution (740 kBq) and ¼ of this volume (5 µL) were diluted in 2 mL of a sucrose/PBS solution (1/4 *v*/*v*) and divided into two parts, similar to the liposomal samples. 

Prior to cellular treatment, sample activities were measured using a γ-counter (Hidex automatic γ-counter, Turku, Finland) for 30 s.

For analysis of the [^131^I]I^−^ half-life, samples were kept at 4 °C and incubated at 20 °C for 0.5 h before measurement. The [^131^I]I^−^ half-life was determined by calculating the slope of the linear regression of [^131^I]I^−^ activities vs. time curves considering the standard deviation of each measurement points.

#### 4.2.2. Characterization of Liposomal Size and Zeta Potential Distributions Using Dynamic and Electrophoretic Light Scattering

Both particle size and zeta potential distributions were measured using a Zetasizer Nano ZS (Malvern Instruments, Malvern, UK) equipped with a HeNe laser (633 nm). Scattered laser light was collected at a constant angle of 173°. Prior to measurements, liposome stock solutions were diluted 1/50 with purified and filtrated water (Milli-Q Gradient A10, Merck Millipore, Darmstadt, Germany). All measurements were performed at 20 °C and repeated three times at 1 min intervals. Data were collected from three independently prepared samples and analyzed using the instrument software (DTS from Malvern Instruments). Reported data are the mean peak positions and standard deviations (mean (SD)).

For stability analysis, liposomes were stored at 4 °C. Before measurements, 20 µL of them were incubated at 20 °C for 0.5 h, diluted, and characterized as described above.

### 4.3. Cell Culture

Experiments were performed with the epithelial human breast cancer cell line MDA-MB-231 (ATTC, Manassas, VA, USA) and the Chinese hamster epithelial cell line derived from ovary cells (CHO). Both cell lines were maintained in DMEM-F12 (Sigma-Aldrich) supplemented with 10% fetal bovine serum (FBS) (Thermo Fisher Scientific, Eugene, OR, USA), 10,000 units of penicillin and 10 mg/mL of streptomycin (both Sigma-Aldrich, Taufkirchen, Germany). During cultivation, cells were kept at 37 °C and 5% CO_2_ in a saturated humid atmosphere. Cell density never exceeded 80% confluence. Before cell seeding, glass surfaces (Ø = 3.5 cm Petri dish) were coated with fibronectin (c = 10 µg/mL in PBS) (BD Biosciences, San Jose, CA, USA) for 30 min at 37 °C. 50,000 cells were seeded on the fibronectin-coated dishes and cultured for 24 h prior to liposomal treatment.

Embryonic rat cortical neurons were obtained as described by Abraham et al. (Animal testing license: 84-02.04.2015.A173, LANUV NRW, Germany) [40]. Additionally, a quantity of 250 cells/mm^2^ was seeded in 500 μL of neurobasal media (Thermo Fisher Scientific, Waltham, MA), supplemented with GlutaMAX (Thermo Fisher Scientific, Waltham, MA, USA), B-27 (Thermo Fisher Scientific, Waltham, MA, USA), and Gentamicin (Sigma-Aldrich, Taufkirchen, Germany). Cells were cultivated over 5 days before treatment.

### 4.4. Incubation of Cells with Liposomes Loaded with [^131^I]I^−^

Fusogenic and endocytic liposomes were centrifuged and used as pellet and supernatant. MDA-MB-231, CHO cells, as well as primary neurons were treated with both fractions for 5 min at 37 °C on a warming plate (VWR, Darmstadt, Germany). As a control, cells were incubated with free [^131^I]I^−^ in phosphate buffered saline (PBS) (Sigma-Aldrich, Taufkirchen, Germany). Prior to the activity measurement of cellular [^131^I]I, they were detached from the surfaces with a 0.05% trypsin–EDTA solution (Sigma-Aldrich, Taufkirchen, Germany) for 5 min. Subsequently, cells were collected in fresh media and sample activities were measured using a γ-counter (Hidex automatic γ-counter, Turku, Finland) for 5 min.

Experiments using human blood materials were performed in accordance with protocols approved by our Institutional Review Board (IRB 9555). The project was reviewed by the same board. The voluntary donors gave written informed consents. For blood cell treatment, 60 µL of fresh human blood was collected from a healthy volunteer by pricking the finger and diluted by adding 60 µL of PBS. The samples were treated in the same way as described above (liposomal pellets, liposomal supernatants, and [^131^I]I^−^ solutions were incubated with blood for 5 min at 37 °C). After treatment, cells were centrifuged at 1500× g (centrifuge 5417R from Eppendorf, Wesseling-Berzdorf, Germany) at room temperature for 5 min and sample activities were measured using a γ-counter (Hidex automatic γ-counter, Turku, Finland) for 5 min.

### 4.5. Incubation of MDA-MB-231 Cells and Human Blood Cells with Liposomes Loaded with Non-Radioactive [^127^I]I^−^

For microscopy imaging of MDA-MB-231, cell nuclei were stained for 15 min with DAPI (blue fluorescence, Eugene, OR, USA), according to the manufacturer’s protocols. Before treatment, the DAPI solution was replaced by 2 mL of fresh medium and cells were incubated for 15 min at 37 °C. Both MDA-MB-231 cells and blood cells were treated with liposomal pellet and supernatant after centrifugation of 1 mL sugar/PBS solution containing [^127^I]I^−^ for 5 min at 37 °C. After treatment of the MDA-MB-231 cells, liposome solutions were replaced by fresh medium and the internalized fluorescence was analyzed by laser scanning microscopy.

### 4.6. Microscopy

Samples were imaged using a confocal laser scanning microscope (LSM 710 from Carl Zeiss MicroImaging GmbH, Jena, Germany) equipped with a near UV laser (405 nm), and a HeNe laser (633 nm). The nuclei staining signal was excited by 405 nm and the emission was detected through the band-pass filter BP430/40 nm. The fluorescence membrane tracer DiR was excited using the 633 nm laser line and the emitted signal was collected through the long-pass filter LP650 nm. For imaging, a plan apochromat 40×/1.40 Ph3 (Carl Zeiss MicroImaging GmbH, Jena, Germany) objective was used. To maintain appropriate culture conditions, the microscope was equipped with an incubator (incubator XL 2, Carl Zeiss MicroImaging GmbH, Jena, Germany). Temperature and CO_2_ were kept constant at 37 °C and 5%, respectively. The experiments were repeated at least three times.

### 4.7. Statistical Analysis

Statistical analyses of data were performed by one-way ANOVA using Origin 9.0 (OriginLab Co., Northampton, MA, USA). Statistical significances were considered as follows: *p* < 0.001 (***), *p* < 0.01 (**), *p* < 0.05 (*).

## 5. Conclusions

Cationic fusogenic liposomes with high membrane fusion ability are well suited for the delivery of the radionuclide ^131^I into breast cancer cells in vitro. The negatively charged iodide ions are strongly bound to the cationic liposomal surface without reducing their fusion capacity, and are delivered directly to the breast cancer cells, while red blood cells remained unaffected. Our results indicate the application potential of cationic liposomes for radiotherapy in nuclear medicine.

## Figures and Tables

**Table 1:** Average hydrodynamic diameter (d), polydispersity index (PDI), and zeta potential (ζ) of the cationic fusogenic (FL) and the control endocytic liposomes (EL) with and without [^127^I]I^−^ cargo. Data are presented as mean (SD). N = 3–5.

Sample	d (SD (nm)	PDI	ζ (SD (mV)
**FL**	376 (5)	0.4	88 (5)
**FL/** **^127^** **I**	456 (51)	0.5	89 (3)
**EL**	2221 (1860)	1.0	−2 (1)
**EL/^127^** **I**	2616 (429)	0.6	−6 (6)

**Table 2:** Relative changes of the hydrodynamic diameter (d), polydispersity index (PDI), and zeta potential (ζ) of the cationic fusogenic (FL) and the control endocytic liposomes (EL) with and without [^127^I]I^−^ cargo during 10 days of storage. Data presented in % as mean (SD). N = 3.

Title	Title	Relative Changes of Hydrodynamic Diameter (%)	Title	Title
**Time (day)**	**FL**	**FL/** **^127^** **I**	**EL**	**EL/^127^** **I**
**1**	100 (1)	100 (11)	100 (84)	100 (16)
**2**	79 (34)	96 (51)	122 (34)	138 (84)
**4**	89 (60)	95 (64)	303 (113)	132 (36)
**10**	82 (48)	79 (27)	115 (42)	90 (27)
		**Relative changes of PDI (%)**		
**Time (day)**	**FL**	**FL/** **^127^** **I**	**EL**	**EL/^127^** **I**
**1**	100 (10)	100 (11)	100 (20)	100 (16)
**2**	114 (31)	100 (28)	70 (26)	105 (33)
**4**	93 (22)	67 (15)	70 (54)	82 (54)
**10**	118 (8)	78 (19)	73 (36)	93 (29)
		**Relative changes of zeta potential (%)**		
**Time (day)**	**FL**	**FL/** **^127^** **I**	**EL**	**EL/^127^** **I**
**1**	100 (6)	100 (3)	100 (50)	100 (100)
**2**	98 (8)	91 (11)	92 (76)	167 (91)
**4**	98 (10)	82 (9)	−117 (22)	−266 (37)
**10**	102 (12)	92 (9)	44 (125)	167 (75)

**Table 3:** Half-life (t_1/2_) of [^131^I]I^−^ measured in different environments. Data presented as mean (SD). N = 3. Note, *p* < 0.05 (*), n.s.—non-significant compared to the [131I]I− in PBS sample.

Environment	t _1/2_ (SD (day)	*p*
**PBS**	12.2 (1.0)	-
**FL_p_**	9.8 (1.0)	*
**FL_s_**	12.9 (1.8)	n.s.
**EL_p_**	11.0 (0.8)	*
**EL_s_**	12.0 (0.5)	n.s.

## Data Availability

The data presented in this study are available on request from the corresponding author.
